# Unveiling Dynamic Hotspots in Protein–Ligand Binding: Accelerating Target and Drug Discovery Approaches

**DOI:** 10.3390/ijms26093971

**Published:** 2025-04-23

**Authors:** Alfonso Trezza, Anna Visibelli, Bianca Roncaglia, Roberta Barletta, Stefania Iannielli, Linta Mahboob, Ottavia Spiga, Annalisa Santucci

**Affiliations:** 1 ONE-HEALTH Laboratory, Department of Biotechnology Chemistry Pharmacy, University of Siena, Via Aldo Moro, 2, 53100 Siena, Italy; anna.visibelli2@unisi.it (A.V.); bianca.roncaglia@unisi.it (B.R.); r.barletta@student.unisi.it (R.B.); stefania.ianniell@student.unisi.it (S.I.); l.mahboob@student.unisi.it (L.M.); ottavia.spiga@unisi.it (O.S.); annalisa.santucci@unisi.it (A.S.); 2 SienabioACTIVE, University of Siena, Via Aldo Moro, 2, 53100 Siena, Italy; 3 MetabERN, University of Siena, Via Aldo Moro, 2, 53100 Siena, Italy

**Keywords:** molecular dynamics simulations, statistical analyses, target–drug discovery, molecular descriptor, target–ligand binding, binding dynamic hotspot

## Abstract

Computational methods have transformed target and drug discovery, significantly accelerating the identification of biological targets and lead compounds. Despite its limitations, in silico molecular docking represents a foundational tool. Molecular Dynamics (MD) simulations, employing accurate force fields, provide near-realistic insights into a compound’s behavior within a biological target. However, docking and MD predictions may be unreliable without precise knowledge of the target binding site. Through MD simulations, we investigated 100 co-crystal structures of biological targets complexed with active compounds, identifying key structural and energy dynamic features that govern target–ligand interactions. Our analysis provides a detailed quantitative description of these parameters, offering critical validation for improving the predictive reliability of docking and MD simulations. This work provides a robust framework for refining early-stage drug discovery and target identification.

## 1. Introduction

Recent advancements in bioinformatics and computational biochemistry have significantly improved our understanding of protein–ligand interactions, a key step in drug discovery [1]. These interactions are crucial for designing small molecules that modulate protein functions and regulate numerous biological processes [2]. Despite this significant progress, accurately predicting protein–ligand interactions remains a major challenge due to protein structures’ dynamic and complex nature [3]. To address these challenges, a variety of computational tools have been developed, including molecular dynamics (MD) simulations, machine learning (ML) algorithms, and advanced molecular docking techniques [4]. Initially, traditional molecular docking predicted how a ligand binds to a protein assuming rigid protein structures, limiting their ability to account for flexibility [5]. However, docking methods have since evolved to account for protein dynamics [6]. Modern techniques, such as flexible and ensemble docking, allow consideration of multiple protein conformations, enhancing the accuracy of binding predictions by better simulating the natural conformational changes proteins undergo, which are crucial for ligand binding [7]. One of the most significant advances in computational biochemistry is the incorporation of chemical and physical descriptors into binding affinity models [8]. Techniques like machine learning [9], free energy perturbation (FEP), and thermodynamic integration (TI) offer more precise predictions by considering complex factors such as solvation effects, entropy, and induced fit mechanisms [10,11]. These methods capture the energetic dynamics of protein–ligand interactions more accurately than traditional scoring functions, leading to more reliable predictions [12,13,14]. These advancements allow for more effective identification of critical parameters, such as hydrogen bonding networks, which are essential for determining the stability and specificity of protein–ligand complexes [15]. However, significant challenges remain, particularly due to the inherent complexity and dynamic nature of protein structures [16]. Proteins undergo conformational changes that significantly affect ligand binding, and many proteins have cryptic or allosteric binding sites that are not visible from static structures [17]. Such sites may only become accessible under specific physiological conditions when the protein adopts alternative conformations, making accurate binding predictions difficult [18]. As a result, resources may be wasted and valuable opportunities in drug discovery missed [19]. Furthermore, despite several structural and chemical–physical properties being found to be involved in target–ligand interactions, a precise numerical description to rationalize the accuracy of docking and MD predictions is currently lacking [20]. This study analyzed the structural and dynamic properties of key binding residues in 100 target–small molecule complexes obtained by the high-resolution X-ray crystallography method available in RCSB Protein Data Bank [21]. By integrating MD simulations with statistical analyses, we identified and quantitatively described the structural and physicochemical parameters essential for ligand recognition, binding, and stability [22]. This approach provides novel guidelines to steer and reinforce the accuracy and reliability of the predicted binding poses [23]. The identification of dynamic ”hotspots” holds significant implications for improving protein–ligand interaction predictions, offering a deeper understanding of ligand recognition and binding, leading to more reliable predictions in drug discovery [24]. Furthermore, identifying dynamic binding residues opens new possibilities for targeting previously overlooked sites, such as allosteric and cryptic binding sites, which may be crucial for therapeutic interventions [25]. By addressing key challenges like protein flexibility and scoring inaccuracies, this methodology enhances predictions of binding sites and poses, ultimately improving the identification of potential lead compounds [26]. As computational techniques continue to advance, the insights gained from these dynamic hotspots will play a central role in refining drug discovery pipelines and expanding the range of therapeutic targets available for modulation [27]. These advancements promise to increase the success rates of high-throughput screening and the rational design of more effective drug-like molecules [28].

## 2. Results

A rigorous selection process was employed to ensure that the chosen co-crystal structures provided a realistic and unbiased foundation for molecular dynamics simulations. The criteria adopted aimed to minimize structural and energetic biases while maintaining consistency across the dataset.

First, only X-ray crystal structures in absence of mutations with atomic-level resolution were considered to ensure the highest accuracy in the representation of protein–ligand interactions. This approach reduces uncertainties associated with lower-resolution structures and allows for a more precise characterization of binding events.

Second, we prioritized soluble proteins to maintain homogeneity in the dataset. The inclusion of only soluble targets minimizes potential variability arising from differences in protein stability and solvent interactions, thereby enabling a more controlled and comparable assessment of ligand–binding dynamics.

Third, only protein–ligand complexes with experimentally confirmed inhibitory activity were included. This criterion ensures that all ligands are biologically relevant and actively participate in binding interactions, reinforcing the physiological significance of the selected structures.

Furthermore, to enhance dataset consistency and streamline computational processing, ligands with complex chemical features, such as those containing ions or unusual functional groups, were excluded. Since such ligands often require additional charge calculations or parameterization steps, their exclusion ensured that all ligands could be treated using standard force field parameters without the need for extensive modifications.

The parameterization of selected ligands followed established protocols, as detailed in the Methods section. By applying these stringent selection criteria, we aimed to create a dataset that was both representative of biologically relevant interactions and optimized for computational efficiency.

### 2.1. Root Mean Square Deviation (RMSD) and Solvent-Accessible Surface Area (SASA)

Root Mean Square Deviation (RMSD) analyses were performed for each system considering the protein backbone, binding residue backbone, and ligand. Firstly, protein backbone RMSD and of the docking poses of all 100 complexes showed a stable trend along the entire MD run with values between 1.5 Å to 4 Å (for the backbone) and 0.14 Å to 3.74 Å (for the docking poses) (Figure S1), respectively, suggesting good structural stability and the reliability of cMD protocols.

**Figure 1:**
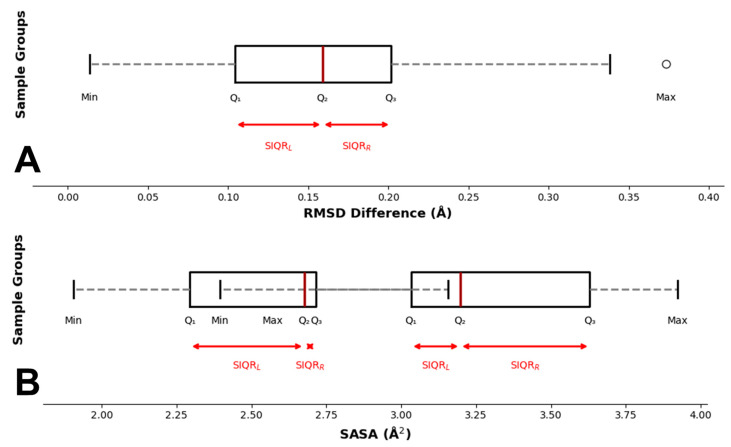
Box plot representations of 100 protein–ligand complexes. (**A**) RMSD and (**B**) SASA box plots reported the values along the MD run. The median is represented as a red line. SIQR represents “Semi-Interquartile Range Left” (SIQR_L_)—the distance between the first quartile (Q1) and the median (Q2). “Semi-Interquartile Range Right” (SIQR_R_)—the distance between the median quartile (Q2) and the third quartile (Q3). The white circle represents the only outlier obtained from our analyses.

Then, two box plots were evaluated (Figure 1A,B), providing a comparative visualization of the residue’s and ligand’s RMSD fluctuations, highlighting their stability throughout the simulations. The RMSD fluctuations of the binding residue backbone revealed a median value of 1.2 Å. The 25th percentile was 0.7 Å, while the 75th percentile reached 1.5 Å, yielding an IQR of 0.8 Å (Figure S2). Similarly, the fluctuations in ligand RMSD values showed a median of 1.6 Å, with the 25th percentile at 1 Å and the 75th percentile at 2 Å, resulting in an interquartile range (IQR) of 1 Å (Figure 1A).

We evaluated the range of the minimum and maximum SASA for all binding residues of the target/ligand complexes. Minimum SASA analysis showed a median of 2.68 Å^2^, with the 25th percentile at 2.29 Å^2^ and the 75th percentile at 2.72 Å^2^, resulting in an interquartile range (IQR) of 0.43 Å^2^. Maximum SASA analysis reported a median value of 3.2 Å^2^. The 25th percentile was 3.03 Å^2^, while the 75th percentile reached 3.62 Å^2^, yielding an IQR of 0.6 Å^2^. From SASA analysis, we evaluated the range of the minimum and maximum SASA for all binding residues of the target–ligand complexes. Minimum SASA analysis showed a median of 2.68 Å^2^, with the 25th percentile at 2.29 Å^2^ and the 75th percentile at 2.72 Å^2^, resulting in an interquartile range (IQR) of 0.43 Å^2^. Maximum SASA analysis reported a median value of 3.2 Å^2^. The 25th percentile was 3.03 Å^2^, while the 75th percentile reached 3.62 Å^2^, yielding an IQR of 0.59 Å^2^. The overall minimum and maximum SASA values obtained are 1.9 Å^2^ and 3.92 Å^2^, respectively (Figure 1B).

### 2.2. H-Bond Occupancy Analyses

**Figure 2:**
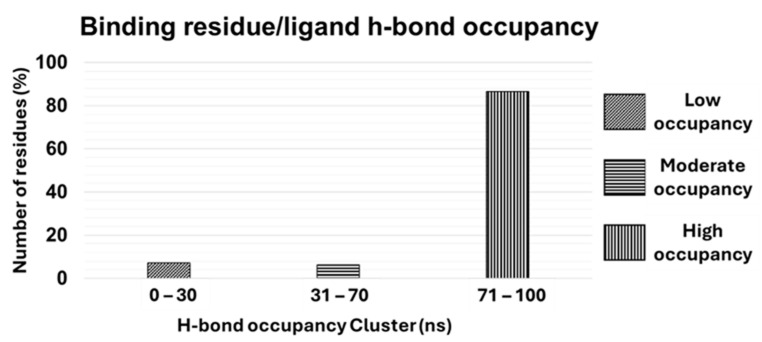
Binding residue–ligand H-bond occupancy.

The target binding residue–ligand H-bonds and the occupancy were analyzed for all complexes with the “gmx bond” function implemented in GROMACS2019.3. Shortly, the existence matrix considered two groups, a donor, and an acceptor, and it showed the existence time of H-bonds formed between a single residue and the ligand along the entire MD run. The existence map was represented by a Cartesian graph where the y and x axes reported the H-bond index (donor/acceptor atom number involved in the interaction) and the time of the MD run, respectively. To standardize our analyses, the existence time was divided into three time clusters: 0 ns–30 ns (low occupancy), 31 ns–70 ns (moderate occupancy), and 71 ns–100 ns (high occupancy). From cMD and statistical analyses, we observed that 23 binding residues belonged to all complexes considered in this study (7.2% of total) fell in the “low occupancy” cluster (from 0 to 30 ns), 20 residues (6.3% of total) in the “moderate occupancy” cluster (from 31 ns to 70 ns), and 275 residues (86.5% of total) in the “high occupancy” cluster (from 71 ns to 100 ns) (Figure 2).

### 2.3. Residue Frequency Forming the Target Binding Pocket

**Figure 3:**
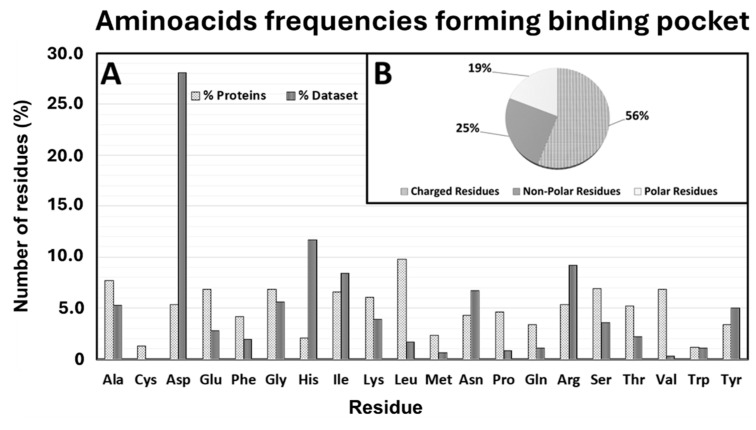
Amino acid frequencies forming binding pockets. (**A**) relative abundance of each residue (white tower) in the entire set of proteins (black tower). (**B**) Frequency of the residues based on their chemical and physical properties.

The analysis of residue frequency within the protein binding pockets (defined as the residues involved in interactions with the ligand) revealed a uniform distribution among most residues. However, certain residues, namely aspartate (28.1%), histidine (11.7%), and arginine (9.2%) exhibited a significantly higher frequency compared to others (Figure 3A). When we clustered these residues based on their chemical and physical properties, an intriguing pattern emerged: charged residues (both positively and negatively charged) accounted for more than half of all residues within the binding pockets (56%). In contrast, non-polar and polar uncharged residues were less frequent, comprising 25% and 19% of the binding pocket residues, respectively (Figure 3B). To ensure the robustness of our findings and avoid potential biases caused by differences in residue composition across the target proteins considered in this study, a statistical analysis was conducted to determine the relative abundance of each residue in the entire set of proteins. This was then compared to the frequency of residues within the binding pockets. Strikingly, the statistical analysis revealed that the residues most frequently involved in the binding pockets—such as aspartate, histidine, and arginine—were among the least abundant in the overall protein composition (Figure 3B).

## 3. Discussion

The results presented in this study offer valuable insights into the structural stability and dynamics of protein–ligand complexes, with a particular focus on binding pocket residues and their role in ligand recognition and stability.

Several structural and energy analyses were explored and evaluated in this study, but only the binding residues and ligand RMSD, binding residues SASA, binding residues/ligand H-bond occupancy, and the frequency of the residues forming the binding pocket provided strong evidence of their involvement in the target–ligand interaction. The Root Mean Square Deviation (RMSD) analyses revealed high structural stability across all protein systems, as indicated by consistently low backbone RMSD values throughout the MD simulations. These values, ranging from 0.15 Å^2^ to 0.4 Å^2^, confirmed the robustness of cMD protocols. The binding residue backbone RMSD also showed tight fluctuation ranges, with an interquartile range (IQR) of 0.08 Å^2^, further supporting the structural integrity of the binding pocket throughout the simulations. Interestingly, the ligand RMSD values showed a median value of 1.6 Å^2^ and an IQR of 1 Å^2^. This suggests that, despite the different structural and chemical-physical proprieties of ligands in complex with binding pockets characterized by different structural and chemical–physical proprieties, the ligands maintained similar binding pose stability within the target binding pocket as shown from the significantly low value of IQR providing evidences that this range can be considered a representative metric to drive the accuracy of the binding pose. Similarly, the Solvent-Accessible Surface Area (SASA) analyses showed minimal fluctuation across the binding residues, indicating that the pocket residues remained well-structured and solvent exposed throughout the MD run. The narrow fluctuation range of 1.91 Å^2^ (2.68 Å^2^ to 3.92 Å^2^), provides a key reference range for assessing a rational binding pose. Hydrogen bond occupancy analyses provided further evidence of the stable nature of these protein–ligand interactions. Most of the binding residues (86.5%) were classified in the “high occupancy” cluster (71 ns) throughout the simulation, indicating that the hydrogen bonds between the ligand and binding residues were maintained consistently over time. This high occupancy reinforces the idea that the ligands were well-docked within the binding pockets and interacted with specific residues sustainably, contributing to the overall stability and specificity of the interaction. Notably, only a small fraction of the residues (7.2%) showed low occupancy, suggesting that transient interactions played a minor role in these complexes as well as in the stability of the ligand within the binding pocket. These findings emphasize the importance of stable, high-occupancy hydrogen bonds in driving ligand binding and stability. One of the most interesting results came from the analysis of residue frequency within the protein binding pockets. Charged residues, such as aspartate (28.1%), histidine (11.7%), and arginine (9.2%), were significantly more frequent in the binding pockets compared to other residues. This observation was further reinforced when residues were categorized by their chemical and physical properties, revealing that more than half (56%) of the binding pocket residues carried a net charge. The prevalence of charged residues in binding pockets suggests that electrostatic interactions play a fundamental role in ligand binding. Charged residues are known to engage in strong interactions with ligands, including ionic interactions and hydrogen bonds, which are vital for ligand recognition and affinity. Moreover, the ability of charged residues to engage in long-range interactions may enhance the likelihood of ligand binding, particularly in the early stages of recognition. In contrast, non-polar and polar uncharged residues were underrepresented in the binding pockets, comprising only 25% and 19% of the residues, respectively. While non-polar residues may contribute to the hydrophobic core of the binding pocket, their limited presence suggests that hydrophobic interactions may play a secondary role compared to electrostatic and hydrogen bonding interactions in stabilizing the ligand. This imbalance highlights the pivotal role of charged residues in dictating the specificity and strength of the protein–ligand interaction. To address potential biases in the residue composition, a comparative statistical analysis was carried out between the overall protein composition and the residues forming the binding pockets. Strikingly, the residues most frequently involved in the binding pockets, particularly aspartate, histidine, and arginine, were among the least abundant in the overall protein composition. This finding suggests that these residues are selectively enriched within binding pockets, playing specialized roles in ligand recognition and binding. Their relative scarcity in the overall protein structure, but high prevalence in binding sites, indicates that they are not randomly distributed but are strategically positioned to enhance the functionality of the binding pocket. The presence of these residues is likely essential for stabilizing the ligand interaction, and their enrichment underscores their importance in the formation and maintenance of a functional binding site. This evidence underlies the critical role of these charged residues in the forming binding sites and highlights their essential contribution to ligand recognition, binding, and stability. The implications of these findings are substantial. This study highlights the critical role of charged residues in mediating these interactions by identifying the structural and chemical properties that contribute to the formation of stable and specific protein–ligand interactions. The selective enrichment of aspartate, histidine, and arginine within binding pockets suggests that these residues are key to designing ligands that can effectively target protein binding sites. Furthermore, the robustness of the MD simulations, as indicated by the stability of the protein backbones and the high occupancy of hydrogen bonds, supports the use of MD as a powerful tool for studying protein–ligand interactions in a dynamic environment.

Importantly, the presence of charged amino acids, such as Asp, Arg, and His, in protein–ligand binding sites is already a well-established observation that can be inferred from structural data available in the Protein Data Bank (PDB) without the use of in silico simulations. However, the primary goal of our analysis was not to reaffirm this known fact but to provide a quantitative measure of how frequently these charged residues contribute to the formation of binding sites across a diverse set of protein-ligand complexes.

By analyzing 100 co-crystal structures, we determined the numerical range and frequency with which these charged residues appear within ligand-binding pockets. This provides a statistically relevant distribution of amino acid composition, offering more than just a qualitative observation. Instead, we present explicit numerical data that can serve as a useful reference in computational drug design.

The quantitative nature of this analysis is particularly valuable in early-stage drug discovery and rational docking studies, especially for targets where no prior binding site information is available. In such cases, having a numerical benchmark for the frequency of charged residues in binding sites can assist in ligand placement, improve scoring functions, and help identify potential binding regions. This numerical characterization offers a precise metric that enhances the reliability of binding site predictions and supports the selection of suitable binding pockets for further investigation.

One of the most striking outcomes of this extended analysis is the consistency observed in the numerical ranges of RMSD, SASA, and hydrogen bond occupancy values, despite the biochemical diversity of the selected systems. Although the dataset includes a predominant number of hydrolases (52%), this reflects the availability of high-quality co-crystallized complexes with experimentally validated ligands rather than an intentional bias. Crucially, the protein and ligand structures sampled cover a wide spectrum of fold classes, binding site geometries, and chemotypes. The fact that all these structurally and functionally distinct systems fall within a relatively narrow window of dynamic and interaction descriptors is of particular significance. It strongly suggests that these parameters—especially RMSD—may serve as reliable, generalizable metrics to assess the stability and plausibility of ligand poses in binding site predictions. This finding reinforces the conceptual core of our work: the identification of consistent quantitative benchmarks capable of guiding pose selection and binding validation, independently of the protein class or ligand type. In this light, our study not only provides a robust and statistically validated dataset but also proposes a novel framework in which the convergence of specific structural descriptors could inform automated and interpretable scoring functions for ligand stability assessment.

In conclusion, this study provides a deeper understanding of the molecular determinants that govern protein–ligand interactions. The identification of charged residues as key players in binding site formation and ligand stabilization offers valuable insights for future drug design efforts. By focusing on the dynamics of these interactions, particularly the role of high-occupancy hydrogen bonds and charged residues, researchers can refine their strategies for predicting binding sites and designing ligands with improved specificity and affinity. Increasing the number of crystal structures could improve the generalizability of the results, and we are open to this in future studies. These findings contribute to overcoming the limitations of current computational models and hold great promise for enhancing the accuracy of drug discovery pipelines.

## 4. Materials and Methods

### 4.1. Structure Resource and Preparation

Three-dimensional structures and FASTA sequences of 100 complexes used in this study were retrieved from the RCSB Protein Data Bank [21] and UniProtKB [29] (Table S1), respectively. To resolve errors during the molecular dynamic simulations, possible missing side chains and steric clashes in PDB files were added and optimized through molecular modeling, carried out using PyMOD3.0 [30]. CLUSTALO 1.2.0 [31] performed the sequence alignments, and Modeller v.9.3 [32] was used as the molecular modeling tool.

To further clarify, no docking simulations were performed. Co-crystal structures were analyzed through molecular dynamics simulations. Prior to the simulations, structural optimization of the complexes was conducted to refine the geometry of the proteins and ligands, without altering the initial docking pose or interactions. The optimization process focused solely on adjusting the structures and adding missing side chains, ensuring they were suitable for accurate molecular dynamics simulations. No parallel docking simulations or pose rearrangements were performed.

All optimized structurally complexes underwent energy minimization to solve potential high-energy intramolecular interactions using GROMACS 2019.3 [33,34] with a charmm36-mar2019 force field [35]. Structures were immersed in a cubic box filled with TIP3P water molecules [36], and counterions were added to balance the net charge of the system. Simulation runs were carried out applying periodic boundary conditions. The system energy was minimized with 5000 steps of minimization with the steepest descent algorithm, converging to minimum energy with forces lower than 100 kJ/mol/nm. The temperature and pressure stayed constant at 300 K and 1 atm using a V-rescale thermostat and Nosé–Hoover barostat, with low dumping of 1 ps^−1^, respectively [37]. The LINCS algorithm constrained the bond lengths involving hydrogen atoms [38]. To relax the system, a short 10 ns cMD was performed, and, consequently, each system was analyzed by the PLIP tool [39] to identify all the protein residues involved in binding with the ligand. Based on PLIP 2.3.0 results, an extra index including the binding residues was generated for each. All parameters to generate the ligand topologies were obtained using CHARMM-GUI 3.8 platform [40]. The system used the “gmx make ndx” function implemented in GROMACS 2019.3; thus, a classical MD (cMD) simulation of 100 ns was run for each biological system.

### 4.2. MD Analyses

The residues involved in H-bonds with the ligand were considered in MD analyses. The RMSD (Root Mean Square Deviation) and SASA (Solvent-Accessible Surface Area) were evaluated for the protein backbone, binding residues, and ligand. Additionally, the target binding residue–ligand interaction occupancy was considered through the existence matrix, implemented in GROMACS 2019.3, with the function “bond” adding the flags-hbn and-hbm. All other analyses were performed by default.

### 4.3. Statistical Analyses

The statistical analyses in this study focused on analyzing the RMSD of binding residues and ligands and SASA of the binding residues across 100 protein–ligand complexes. The extent of fluctuation in the RMSD values over the simulations was computed for each binding residue and ligand, respectively. The range was determined by the difference between the maximum and minimum RMSD values observed for each residue during MD simulations. Additionally, a statistical analysis of SASA has been performed. The range of the minimum SASA values across all binding residues was calculated and graphically analyzed, offering insights into the solvent exposure during ligand binding. The same analysis was made with the range of maximum SASA values, showcasing the variability in solvent exposure of binding residues throughout the MD simulations.

## 5. Conclusions

A novel computational pipeline was set to steer and define the reliability of protein–ligand interactions. Particularly, this in silico approach is designed to optimize the docking simulation reaction environment and help the selection of the best binding pose. Additionally, new structural/physical parameters, evaluated by MD simulations, were proposed as a key insight into the inhibitor binding. High-accuracy dynamics parameters, such as RMSD, SASA, H-bond occupancy, and frequency of residues forming the target binding pocket, were statistically analyzed, adding confidence to the MD simulations between a biological target and an active compound.

It is important to emphasize that this study aimed not only to show the already-known features of target–ligand interactions but to provide a numerical description of key features involved in the recognition, binding, and stability of the ligand against the target. The analyses conducted provide a significant and meaningful contribution to the knowledge of ligand docking, particularly in understanding the dynamic and structural features that govern protein–ligand interactions. While the critique suggests that the findings are specific to the systems studied, the methodologies and insights offered by this work have broader implications for the field of computational drug discovery.

The study provides a detailed quantitative description of key structural and energetic parameters that govern protein–ligand interactions, such as RMSD, SASA, and hydrogen bond occupancy. These metrics are not merely descriptive but offer critical benchmarks for evaluating the stability and reliability of docking predictions. The low RMSD values observed for both binding residues and ligands across 100 complexes demonstrate that the ligands maintain stable binding poses within the target pockets. This stability is a key indicator of reliable docking predictions and provides a reference range for future docking studies. The narrow fluctuation ranges in SASA values suggest that binding residues remain well-structured and solvent-exposed throughout the simulations. This insight is crucial for understanding how solvent accessibility influences ligand binding and can guide the design of ligands that optimize interactions with solvent-exposed residues.

By establishing these quantitative benchmarks, the study provides a robust framework for validating docking poses and improving the accuracy of computational predictions. These metrics can be applied to other systems to assess the reliability of docking results, making the findings broadly applicable.

One of the most significant contributions of this study is the identification of dynamic hotspots—key residues that play a critical role in ligand recognition and binding. The analysis reveals that charged residues (e.g., aspartate, histidine, and arginine) are disproportionately enriched in binding pockets, accounting for over 56% of the residues involved in ligand interactions. This finding has several important implications such as the potential individuation of allosteric and cryptic sites by analyzing the docking study through these key dynamics parameters, and their quantitative ranges are provided in this study for improving the accuracy of docking predictions and expanding the range of druggable targets.

The analysis of hydrogen bond occupancy reveals that 86.5% of binding residues maintain high-occupancy hydrogen bonds (71–100 ns) with the ligand throughout the simulations. This finding underscores the importance of stable, high-occupancy interactions in driving ligand binding and stability. Key implications include the validation of docking poses considering the high-occupancy hydrogen bonds serve as a critical validation metric for docking poses. Ligands that form stable hydrogen bonds with binding residues are more likely to exhibit strong binding affinity, making this metric a valuable tool for evaluating docking results; thus, an important aspect of our results is the quantification of the hydrogen bond occupancy, and this study provides a novel and practical approach for assessing the stability of protein–ligand interactions, which can be applied to other systems to improve docking accuracy.

In summary, the study provides a comprehensive and rigorous data analysis of protein–ligand interactions, offering valuable insights into the structural and dynamic features that govern ligand binding. By identifying dynamic hotspots, such as RMSD and SASA, quantifying hydrogen bond occupancy, and integrating MD simulations with docking, the study addresses key limitations of traditional docking methods and provides a robust framework for improving the accuracy and reliability of docking predictions when no information is available. The findings are not only relevant to the specific systems studied but also have broad applicability to other protein–ligand interactions, making a significant contribution to the knowledge of ligand docking and computational drug discovery.

Altogether, this work suggests a novel numerical range of dynamics molecular descriptors and offers a comprehensive computational framework to accelerate biological target druggable pocket identification and drug discovery approaches.

## Data Availability

The original contributions presented in this study are included in the article. Further inquiries can be directed to the corresponding author.

## Supplementary Materials

The supporting information can be downloaded at: https://www.mdpi.com/article/10.3390/ijms26093971/s1.
