# Supplementary material for: Unveiling Dynamic Hotspots in Protein–Ligand Binding: Accelerating Target and Drug Discovery Approaches
Source: Int J Mol Sci. 2025 Apr 23;26(9):3971. doi: 10.3390/ijms26093971 (PMC12071544; doi:10.3390/ijms26093971)
Supplement: Supplementary file 1 [file ijms-26-03971-s001.zip › Table S1.pdf]

# Unveiling Dynamic Hotspots in Protein-Ligand Binding: Accelerating Target and Drug Discovery Approaches

Alfonso Trezza[1,*], Anna Visibelli[1], Bianca Roncaglia[1], Roberta Barletta[1], Stefania Iannielli[1], Linta Mahboob[1], Ottavia Spiga[1], and Annalisa Santucci[1,2,3]

1 ONE-HEALTH Lab, Department of Biotechnology Chemistry Pharmacy, University of Siena, Via Aldo Moro, 2, Siena, 53100, Italy, Siena.

alfonso.trezza2@unisi.it; anna.visibelli2@unisi.it; bianca.roncaglia@unisi.it; r.barletta@student.unisi.it; stefania.ianniell@student.unisi.it;

l.mahboob@student.unisi.it; ottavia.spiga@unisi.it; annalisa.santucci@unisi.it

2 SienabioACTIVE, University of Siena, Via Aldo Moro, 2, Siena, 53100, Italy, Siena. annalisa.santucci@unisi.it

3 MetabERN, University of Siena, Via Aldo Moro, 2, Siena, 53100, Italy, Siena. annalisa.santucci@unisi.it

*Correspondence: alfonso.trezza2@unisi.it

| CLASSIFICATION | UniProtKB | PDB Code | Method | Resolution (Å) |
|---|---|---|---|---|
| **HYDROLASE** | P22894 | 1A85 | X-ray | 2.00 |
| **HYDROLASE** | P22894 | 1A86 | X-ray | 2.00 |
| **ASPARTYL PROTEASE** | P03366 | 1AJV | X-ray | 2.00 |
| **ASPARTYL PROTEASE** | P03366 | 1AJX | X-ray | 2.00 |
| **SERINE PROTEASE** | P00760 | 1AZ8 | X-ray | 1.80 |
| **HYDROLASE** | P08254 | 1B8Y | X-ray | 2.00 |
| **HYDROLASE** | P04587 | 1BDL | X-ray | 2.80 |
| **HYDROLASE** | P08254 | 1CAQ | X-ray | 1.80 |
| **METALLOPROTEINASE** | P08254 | 1CIZ | X-ray | 1.64 |
| **HYDROLASE** | P03366 | 1D4I | X-ray | 1.81 |
| **HYDROLASE** | P03367 | 1DCY | X-ray | 2.70 |
| **HYDROLASE** | P03368 | 1EBW | X-ray | 1.81 |
| **HYDROLASE** | P03369 | 1EC0 | X-ray | 1.79 |
| **HYDROLASE** | P03370 | 1EC1 | X-ray | 2.10 |
| **HYDROLASE** | P03371 | 1EC3 | X-ray | 1.80 |
| **TRANSFERASE** | P03372 | 1EX8 | X-ray | 1.85 |
| **HYDROLASE** | P03373 | 1FDK | X-ray | 1.91 |
| **HYDROLASE** | P03374 | 1HFS | X-ray | 1.70 |
| **HYDROLASE** | P03375 | 1HTE | X-ray | 2.80 |
| **HYDROLASE** | P03376 | 1JH1 | X-ray | 2.70 |
| **TRANSFERASE** | P03378 | 1JR4 | X-ray | 2.63 |
| **HYDROLASE** | P03379 | 1LO6 | X-ray | 1.56 |
| **HYDROLASE** | P03381 | 1NX3 | X-ray | 2.45 |
| **ASPARTIC PROTEASE** | P03382 | 1ODY | X-ray | 2.00 |
| **HYDROLASE** | P03383 | 1RGK | X-ray | 1.87 |

| | | | | |
|---|---|---|---|---|
| **HYDROLASE** | P03385 | 1SBG | X-ray | 2.30 |
| **HYDROLASE** | P03386 | 1SNC | X-ray | 1.65 |
| **BIOTIN BINDING PROTEIN** | P03387 | 1STP | X-ray | 2.60 |
| **LIGASE** | P03388 | 1T4E | X-ray | 2.60 |
| **HYDROLASE** | P03389 | 1TCW | X-ray | 2.40 |
| **HYDROLASE** | P03390 | 1TCX | X-ray | 2.30 |
| **ASPARTYL PROTEASE** | P03391 | 1VIJ | X-ray | 2.40 |
| **HYDROLASE** | P03392 | 1W5V | X-ray | 1.80 |
| **HYDROLASE** | P03367 | 1ZJ7 | X-ray | 1.93 |
| **HYDROLASE** | O92139 | 1ZP8 | X-ray | 2.02 |
| **HYDROLASE** | P03366 | 1ZPA | X-ray | 2.02 |
| **HYDROLASE** | P22894 | 1ZVX | X-ray | 1.87 |
| **HYDROLASE** | O92139 | 2BQV | X-ray | 2.10 |
| **HYDROLASE** | Q672W7 | 2EW6 | X-ray | 2.20 |
| **HYDROLASE** | P03367 | 2PWC | X-ray | 1.78 |
| **HYDROLASE** | P03367 | 2QNP | X-ray | 1.41 |
| **HYDROLASE** | P03367 | 2QNQ | | |
| **HYDROLASE** | P04587 | 2R3W | X-ray | 1.92 |
| **LYASE** | P56221 | 2STD | | |
| **HYDROLASE** | P08254 | 2USN | X-ray | 2.20 |
| **IMMUNE SYSTEM** | Q7V867 | 2XCZ | X-ray | 1.64 |
| **HYDROLASE** | P09237 | 2Y6C | X-ray | 1.70 |
| **HYDROLASE** | P09237 | 2Y6D | X-ray | 1.60 |
| **HYDROLASE** | P03367 | 2ZGA | X-ray | 1.65 |
| **CHAPERONE** | P07900 | 3BMY | X-ray | 1.60 |
| **HYDROLASE** | P03367 | 3CKT | X-ray | 1.65 |

| | | | | |
|---|---|---|---|---|
| **HYDROLASE** | P39900 | 3EHX | X-ray | 1.90 |
| **HYDROLASE** | P39900 | 3EHY | X-ray | 1.90 |
| **HYDROLASE** | P03369 | 3EL4 | X-ray | 2.00 |
| **LIPID BINDING PROTEIN** | P15090 | 3FR4 | X-ray | 2.16 |
| **LIPID BINDING PROTEIN** | P15090 | 3FR5 | X-ray | 2.20 |
| **ISOMERASE** | C3SLN3 | 3G7E | X-ray | 2.20 |
| **PROTEIN BINDING** | P04117 | 3HK1 | X-ray | 1.70 |
| **HYDROLASE** | P03369 | 3I7E | X-ray | 1.70 |
| **HYDROLASE** | P03367 | 3JVW | X-ray | 1.80 |
| **ISOMERASE** | Q13526 | 3KAF | X-ray | 2.30 |
| **LYASE** | P00918 | 3K34 | X-ray | 0.9 |
| **LIGASE** | Q00987 | 3LBK | X-ray | 2.30 |
| **HYDROLASE** | P39900 | 3LK8 | X-ray | 1.80 |
| **HYDROLASE** | P39900 | 3N2U | X-ray | 1.81 |
| **TRANSFERASE** | P22734 | 3NXT | X-ray | 1.73 |
| **CHAPERONE** | Q4Q4I6 | 3Q5K | X-ray | 2.35 |
| **HYDROLASE** | Q000H7 | 3R0Y | X-ray | 1.65 |
| **TRANSCRIPTION REPRESSOR** | P9WMC1 | 3SDG | X-ray | 1.87 |
| **TRANSCRIPTION REPRESSOR** | P9WMC1 | 3SFI | X-ray | 2.31 |
| **HYDROLASE** | Q92876 | 3VFE | X-ray | 1.88 |
| **IMMUNE SYSTEM** | Q5YD59 | 3ZUI | X-ray | 1.71 |
| **HYDROLASE** | Q8Q3H0 | 4A4Q | X-ray | 1.80 |
| **HYDROLASE** | P00747 | 4CIK | X-ray | 1.78 |
| **TRANSFERASE** | P26281 | 4F7V | X-ray | 1.73 |

| | | | | |
|---|---|---|---|---|
| **HYDROLASE** | P03367 | 4FE6 | X-ray | 2.00 |
| **TRANSFERASE** | P55144 | 4FF8 | X-ray | 2.40 |
| **ASPARTIC PROTEASE** | Q66972 | 4FIV | X-ray | 1.80 |
| **HYDROLASE** | P39900 | 4GR0 | X-ray | 1.50 |
| **Chaperone** | P07900 | 4HY6 | X-ray | 1.65 |
| **HYDROLASE** | P12497 | 4JMU | X-ray | 2.00 |
| **LIGASE** | P56273 | 4JRG | X-ray | 1.90 |
| **LIGASE** | P56273 | 4JSC | X-ray | 2.50 |
| **LIGASE** | Q00987 | 4JV9 | X-ray | 2.50 |
| **LIGASE** | Q00987 | 4JVE | X-ray | 2.30 |
| **TRANSCRIPTION REGULATOR** | A0A0H2Z7 A 6 | 4JVI | X-ray | 2.90 |
| **APOPTOSIS** | P98170 | 4KJU | X-ray | 1.60 |
| **DNA BINDING PROTEIN** | P27694 | 4LUZ | X-ray | 1.90 |
| **CHAPERONE** | P07900 | 4LWH | X-ray | 1.70 |
| **CHAPERONE** | P07900 | 4LWI | X-ray | 1.70 |
| **TRANSCRIPTION REPRESSOR** | P9WMC1 | 4M3B | X-ray | 2.00 |
| **TRANSCRIPTION REPRESSOR** | P9WMC1 | 4M3E | X-ray | 2.11 |
| **TRANSCRIPTION REPRESSOR** | P9WMC1 | 4M3F | X-ray | 2.00 |
| **TRANSCRIPTION REPRESSOR** | P9WMC1 | 4M3G | X-ray | 2.30 |
| **ISOMERASE** | A5K8X6 | 4MGV | X-ray | 1.72 |
| **LIPID BINDING PROTEIN** | O43924 | 5ML3 | X-ray | 1.40 |
| **CELL CYCLE** | P24941 | 6Q4G | X-ray | 0.98 |
| **LYASE** | P00918 | 6T81 | X-ray | 0.98 |
| **LYASE** | P00918 | 7OYN | X-ray | 0.98 |

| | | | | |
|---|---|---|---|---|
| **TRANSFERASE** | P31153 | 7RWG | X-ray | 0.97 |
| **GALACTOSIDE-BINDING LECTIN** | P17931 | 7XFA | X-ray | 0.98 |

**Table S1. Target details**
