# Supplementary figures and images for: Unveiling Dynamic Hotspots in Protein–Ligand Binding: Accelerating Target and Drug Discovery Approaches
Source: Int J Mol Sci. 2025 Apr 23;26(9):3971. doi: 10.3390/ijms26093971 (PMC12071544; doi:10.3390/ijms26093971)

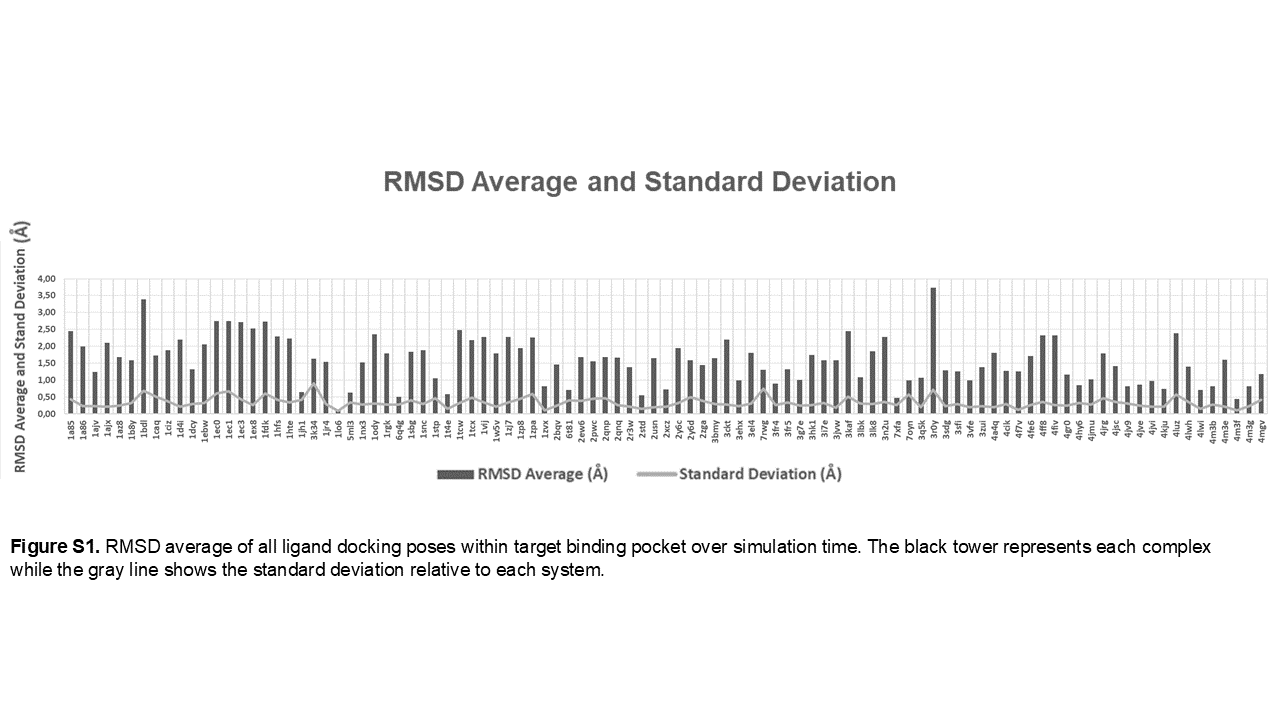

Supplement: Supplementary file 1 [file ijms-26-03971-s001.zip › Figure S1.png]

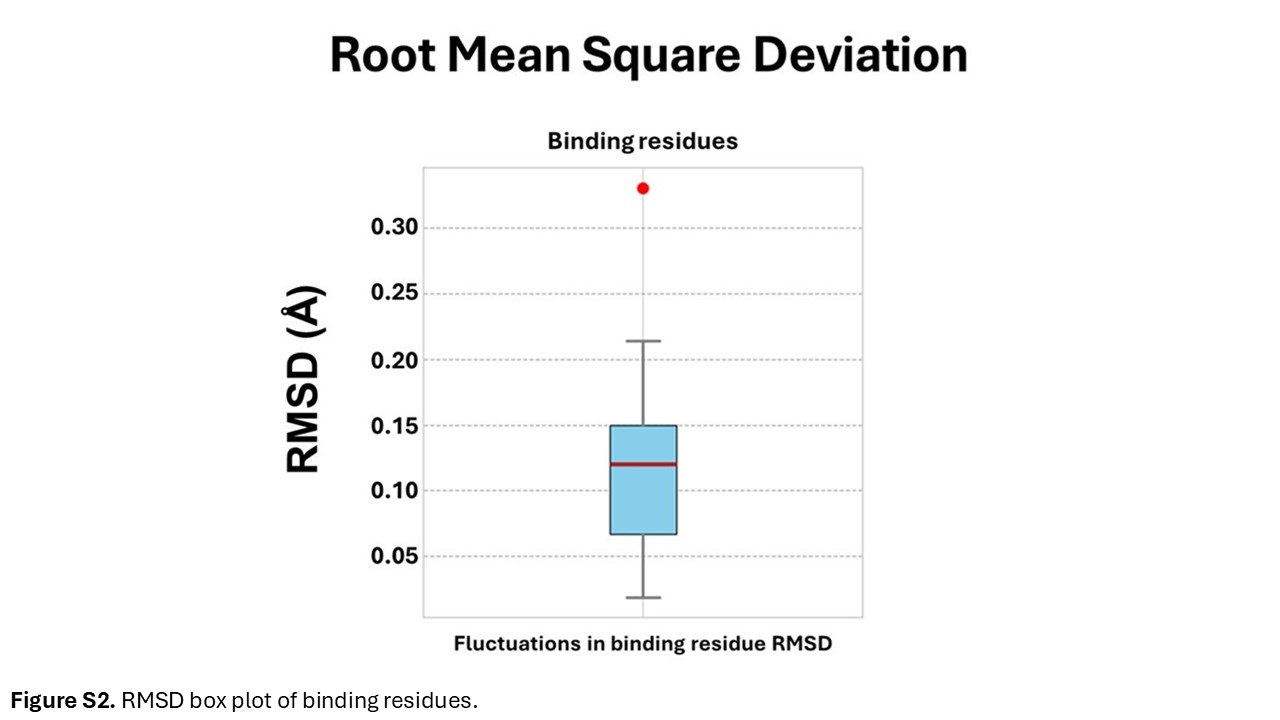

Supplement: Supplementary file 1 [file ijms-26-03971-s001.zip › Figure S2.png]
